# Antibacterial and Herbicidal Activity of Ring-Substituted 3-Hydroxynaphthalene-2-carboxanilides †

**DOI:** 10.3390/molecules18077977

**Published:** 2013-07-08

**Authors:** Jiri Kos, Iveta Zadrazilova, Matus Pesko, Stanislava Keltosova, Jan Tengler, Tomas Gonec, Pavel Bobal, Tereza Kauerova, Michal Oravec, Peter Kollar, Alois Cizek, Katarina Kralova, Josef Jampilek

**Affiliations:** 1 Department of Chemical Drugs, Faculty of Pharmacy, University of Veterinary and Pharmaceutical Sciences, Palackeho 1/3, 612 42 Brno, Czech Republic; 2 Department of Infectious Diseases and Microbiology, Faculty of Veterinary Medicine, University of Veterinary and Pharmaceutical Sciences, Palackeho 1/3, 612 42 Brno, Czech Republic; 3 CEITEC VFU, University of Veterinary and Pharmaceutical Sciences, Palackeho 1/3, 612 42 Brno, Czech Republic; 4 Institute of Chemistry, Faculty of Natural Sciences, Comenius University, Mlynska dolina Ch-2, 842 15 Bratislava, Slovakia; 5 Department of Human Pharmacology and Toxicology, Faculty of Pharmacy, University of Veterinary and Pharmaceutical Sciences, Palackeho 1/3, 612 42 Brno, Czech Republic; 6 Global Change Research Centre AS CR, Belidla 986/4a, 603 00 Brno, Czech Republic

**Keywords:** hydroxynaphthalene-2-carboxanilides, lipophilicity, photosynthetic electron transport inhibition, spinach chloroplasts, *in vitro* antibacterial activity, *in vitro* antimycobacterial activity, *in vitro* cytotoxicity, structure-activity relationships

## Abstract

In this study, a series of twenty-two ring-substituted 3-hydroxy-*N*-phenylnaphthalene-2-carboxanilides were prepared and characterized. The compounds were tested for their activity related to inhibition of photosynthetic electron transport (PET) in spinach (*Spinacia oleracea* L.) chloroplasts. Primary *in vitro* screening of the synthesized compounds was also performed against four *Staphylococcus* strains and against two mycobacterial species. 3-Hydroxy-*N*-(2-methoxyphenyl)naphthalene-2-carboxamide showed high biological activity (MIC = 55.0 µmol/L) against *S. aureus* as well as methicillin-resistant strains. *N*-(2-Fluorophenyl)-3-hydroxynaphthalene-2-carboxamide showed higher activity (MIC = 28.4 µmol/L) against *M. marinum* than the standard isoniazid and 3-hydroxy-*N*-(4-nitrophenyl)naphthalene-2-carboxamide expressed higher activity (MIC = 13.0 µmol/L) against *M. kansasii* than the standard isoniazid. Cytotoxicity assay of effective antimicrobial compounds was performed using the human monocytic leukemia THP-1 cell line. The PET-inhibiting activity expressed by IC_50_ value of the most active compound 3-hydroxy-*N*-(3-nitrophenyl)naphthalene-2-carboxamide was 16.9 μmol/L. The structure-activity relationships of all compounds are discussed.

## 1. Introduction

The increasing number of bacterial, mycobacterial and associated fungal infections underlines the importance of searching for new antimicrobial chemotherapeutics [1]. Tuberculosis and other mycobacterial diseases are common, and in many cases lethal, infectious illnesses caused by various strains of pathogenic mycobacteria. The genus *Mycobacterium* consists of a closely related group of fast and slow-growing species. *Mycobacterium tuberculosis* causes one of the most serious human infections, tuberculosis. Difficulties should be considered while studying *M. tuberculosis*, especially a slow growth rate and the requirement to work in high containment biosafety facilities. To lower risks and make manipulation in the laboratory easier, surrogate model pathogens for *M. tuberculosis* can be used in laboratory studies. M. marinum is very closely related to the *M. tuberculosis*; it is the cause of TB-like infections in poikilothermic organisms, especially frogs and fish. *M. marinum* is a good model for studying because of the lower risk for laboratory workers, genetic relatedness and similar pathology to human TB [2,3]. However, because of *M. tuberculosis,* the pathogenic role of nontuberculous mycobacteria (NTM) in humans was overshadowed for a long time. *M. kansasii*, the most virulent of the NTM, causes nontuberculous mycobacterial lung infections which are very common nowadays and can be indistinguishable from tuberculosis [4]. That is the reason why *M. marinum* and *M. kansasii* are often chosen as model species for screening of prospective antimycobacterial drugs to control mycobacterial diseases.

The treatment of tuberculosis and nontuberculous diseases is mediated by administration of various antimicrobial chemotherapeutics, however massive using of these drugs is considered to be the main reason for increased antibiotic resistance among bacteria [5,6]. The antibiotic resistance of important Gram-positive pathogen, *Staphylococcus aureus*, has become one of the most challenging and persistent worldwide health problems. Methicillin-resistant *S. aureus* (MRSA) was first described in 1961 and since then has become one of the most common clinically relevant bacterial pathogens isolated almost all over the World. Even though originally limited to hospitals, nowadays MRSA is an increasing cause of infections in the community. Recent studies have shown that, despite antibacterial therapy, MRSA infections are still associated with serious clinical consequences (treatment failure, higher morbidity and mortality, prolonged hospitalization, *etc.*). Because of the changing features of MRSA, it is one of the most difficult bacteria for clinicians to treat. The emergence of resistance to currently available drugs, their toxicity and general lack of oral agents justify an urgent need for new anti-MRSA agents [7,8].

Both pharmaceuticals and pesticides are designed to target particular biological functions, and in some cases these functions overlap in their molecular target sites, or they target similar processes or molecules. Taking into consideration that herbicides may also have molecular sites of action in mammals, until recently most pharmaceutical companies had pesticide divisions, sometimes with a different name. All compounds generated by either division of the company were evaluated for both pesticide and pharmaceutical uses. In the past, some leading pesticides have become pharmaceuticals and *vice versa* [9,10,11]. Moreover, good correlation between microbiological activities and herbicidal effects was found [12,13,14,15].

The presence of an amide (-NHCO-) group [16,17,18,19,20] is characteristic of a number of herbicides acting as photosynthesis inhibitors. Over 50% of commercially available herbicides act by reversibly binding to photosystem II (PS II), a membrane-protein complex in the thylakoid membranes, which catalyses the oxidation of water and the reduction of plastoquinone [21], and thereby inhibit photosynthesis [22,23,24].

Promising results of biological screening of some salicylanilides (their antimicrobial, antimycobacterial, antifungal, molluscicidal and herbicidal action) [17,18,19,25,26,27,28,29,30,31,32,33] inspired us to prepare and evaluate ring-substituted 3-hydroxynaphthalene-2-carboxanilides. The design of these 3-hydroxy-*N*-phenylnaphthalene-2-carboxanilides is based on ring analogy with salicylanilides (2-hydroxy-*N*-phenylbenzamide). Thus in the context of the previously-published results [16,17,18,19,20,25,26,27,28,29,30,31,32,33], primary *in vitro* screening of the synthesized compounds was also performed against four *Staphylococcus* strains, three of which were methicillin-resistant *Staphylococcus aureus* strains, and against two mycobacterial species, such as *Mycobacterium marinum* and *M. kansasii*.

## 2. Results and Discussion

### 2.1. Chemistry

**Scheme 1:**
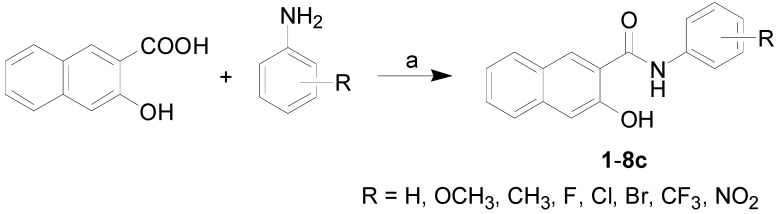
Synthesis of ring-substituted 3-hydroxynaphthalene-2-carboxanilides **1**–**8c**.

All the studied compounds were prepared according to Scheme 1. Modified microwave-assisted synthesis [29] facilitated the process of obtaining 3-hydroxynaphthalene-2-carboxanilides, thus synthesis of the target compounds was carried out only by one step. At first the carboxyl group was activated with phosphorus trichloride. The final amide was immediately formed by aminolysis of the acyl chloride by ring-substituted aniline in dry chlorobenzene. All the compounds were recrystallized from ethanol; their HPLC purity exceeded 97%. By microwave-assisted synthesis reaction times were shortened from hours [34,35,36,37] to minutes, and yields of most compounds (except 2-NO_2_ derivative **8a**) were more than 65%, for details see Section 3.2.

*Reagents and conditions*: (a) PCl_3_, chlorobenzene, MW.

### 2.2. Lipophilicities

**Figure 1:**
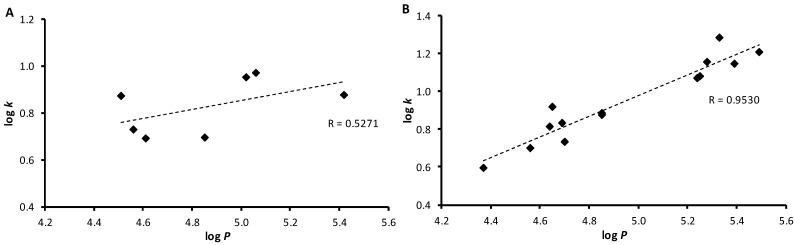
Comparison of experimentally found log *k* values with calculated log *P* of *ortho*-substituted (**A**) and *meta*- and *para*-substituted derivatives (**B**).

Lipophilicity is a property that has a major effect on solubility, absorption, distribution and biotransformation as well as pharmacological activity, because drugs cross biological membranes through passive transport, which strongly depends on their lipophilicity. Lipophilicity has been studied and applied as an important drug property for decades. Lipophilicity of the studied compounds was determined by RP-HPLC as capacity factor logarithm (log *k*) and calculated as log *P* using ACD/Percepta software. The results of ring-substituted 3-hydroxynaphthalene-2-carboxanilides **1**–**8c** are shown in Table 1 and illustrated in Figure 1. The highest experimental lipophilicity was found for 3-hydroxy-*N*-(4-trifluoromethylphenyl)naphthalene-2-carboxamide (**7c**), while 3-hydroxy-*N-*phenylnaphthalene-2-carboxamide (**1**) showed the lowest log *k* value. It is important to note that lipophilicity also has a great impact on target and off-target interactions, as mentioned below.

The results obtained with the discussed compounds show that the experimentally-determined lipophilicities (log *k*) of the *meta*- and *para*-substituted compounds are in accordance with the calculated log *P* values as shown in Figure 1B, while *ortho*-substituted derivatives showed poor match, see Figure 1A. The most significant deviations within the dependence illustrated in Figure 1B can be observed for 4-CF_3_ (**7c**) and 4-NO_2_ (**8c**). The influence of R substituents on lipophilicity is as follows: H < OCH_3_ < CH_3_ < F < NO_2_ < Cl < Br ≤ CF_3_. Within the individual series the lipophilicity determined by log *k* values increases for halogens and methyl substituents as follows: *ortho* < *para* < *meta*; for methoxy substituent as follows: *para* < *ortho* < *meta*, and for CF_3_ and NO_2_ as follows: *ortho* < *meta* < *para*. Generally, it could be concluded that the prediction power of the used experimental log *k* or calculated log *P* values, especially for *meta*- and *para*-substituted derivatives, may be a good tool for searching potential drugs. It can be assumed that log *k* values specify lipophilicity within individual series of the studied compounds.

### 2.3. Inhibition of Photosynthetic Electron Transport (PET) in Spinach Chloroplasts

The activity of the evaluated naphthanilide derivatives related to inhibition of photosynthetic electron transport (PET) in spinach (*Spinacia oleracea* L.) chloroplasts was moderate or low relative to the standard, see Table 1. Generally compounds showed poor aqueous solubility. Only seven compounds from twenty-two tested compounds could be evaluated. PET inhibition by **1**, **2c**–**5c**, **6b**, **6c** and **7b**, **7c** could not be determined due to precipitation of the compounds during the experiments. With respect to these small but specifically substituted groups of compounds some structure-activity relationships (SAR) can be proposed. Compound **8b** (R = 3-NO_2_) expressed the highest PET-inhibiting activity (IC_50_ = 16.9 µmol/L), while compound **8c** (R = 4-NO_2_) expressed the lowest PET-inhibiting activity (IC_50_ = 187.5 µmol/L).

**Table 1:** Structure of ring-substituted 3-hydroxynaphthalene-2-carboxanilides **1**–**8c**, experimentally determined values of lipophilicity log *k*, calculated values of log *P* and electronic Hammett’s σ parameters, IC_50_ [μmol/L] values related to PET inhibition in spinach chloroplasts in comparison with 3-(3,4-dichlorophenyl)-1,1-dimethylurea (DCMU) standard, *in vitro* anti-*Staphylococcus* activities [MIC (μmol/L)] in comparison with standards ampicillin (APC), *in vitro* antimycobacterial activity [MIC (μmol/L)] of compounds **1**–**8c** compared to isoniazid (INH) standard and *in vitro* cytotoxicity assay (LD_50_) of selected compounds.

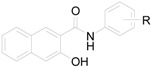												
Comp.	R	log *k*	log *P ^a^*	σ *^a^*	[μmol/L]							
					PET IC_50_	MIC						LD_50_
						SA	MRSA 63718	MRSA 630	MRSA 3202	MM	MK	
**1**	H	0.6310	4.52	0	*^b^*	>972	>972	>972	>972	122	972	–
**2a**	2-OCH_3_	0.6916	4.61	−0.28	59.5	**55.0**	**55.0**	**55.0**	**55.0**	873	218	>30
**2b**	3-OCH_3_	0.6971	4.56	0.12	53.4	>873	>873	>873	>873	>873	**54.6**	>30
**2c**	4-OCH_3_	0.5951	4.37	−0.27	*^b^*	>873	>873	>873	>873	>873	873	–
**3a**	2-CH_3_	0.6936	4.85	−0.17	*^b^*	>923	>923	>923	462	462	115	–
**3b**	3-CH_3_	0.8831	4.85	−0.07	*^b^*	>923	>923	>923	462	923	**57.7**	>30
**3c**	4-CH_3_	0.8753	4.85	−0.17	*^b^*	>923	>923	462	231	>923	923	–
**4a**	2-F	0.7303	4.56	0.06	*^b^*	>910	>910	455	228	**28.4**	**56.9**	>30
**4b**	3-F	0.8296	4.69	0.34	*^b^*	>910	>910	455	228	114	114	–
**4c**	4-F	0.7317	4.70	0.06	*^b^*	>910	>910	455	228	910	114	–
**5a**	2-Cl	0.9509	5.02	0.22	*^b^*	>860	>860	>860	215	860	860	–
**5b**	3-Cl	1.0796	5.25	0.37	*^b^*	>860	>860	430	215	>860	860	–
**5c**	4-Cl	1.0687	5.24	0.23	*^b^*	>860	>860	>860	>860	>860	430	–
**6a**	2-Br	0.9715	5.06	0.22	43.2	>748	>748	>748	>748	>748	748	–
**6b**	3-Br	1.1536	5.39	0.39	*^b^*	>748	>748	>748	187	>748	748	–
**6c**	4-Br	1.1459	5.28	0.23	*^b^*	>748	>748	>748	187	>748	374	–
**7a**	2-CF_3_	0.8762	5.42	0.51	105.2	>773	>773	>773	**97**	193	193	28.6 ± 0.5
**7b**	3-CF_3_	1.2053	5.49	0.43	*^b^*	>748	>748	374	187	>748	748	–
**7c**	4-CF_3_	1.2835	5.33	0.51	*^b^*	>748	>748	374	187	>748	187	–
**8a**	2-NO_2_	0.8710	4.51	0.77	106.6	>830	>830	415	415	830	830	–
**8b**	3-NO_2_	0.8143	4.64	0.71	**16.9**	>830	>830	>830	>830	>830	415	2.5 ± 0.9
**8c**	4-NO_2_	0.9175	4.65	0.78	187.5	>830	>830	>830	>830	>830	**13.0**	<0.37
**DCMU**	–	–	–		1.9	–	–	–	–	–	–	–
**APC**	–	–	–			5.7	>45.8	>45.8	>45.8	–	–	–
**INH**						–	–	–	–	467	29.2	–

*^a^* calculated using sw. ACD/Percepta ver. 2012; *^b^* precipitation during experiment; *SA* = * Staphylococcus aureus* ATCC 29213; MRSA = clinical isolates of methicillin-resistant *S. aureus* 63718, SA 630 and SA 3202 (National Institute of Public Health, Prague, Czech Republic); MM = *Mycobacterium marinum* CAMP 5644, MK = *M. kansasii* DSM 44162.

**Figure 2:**
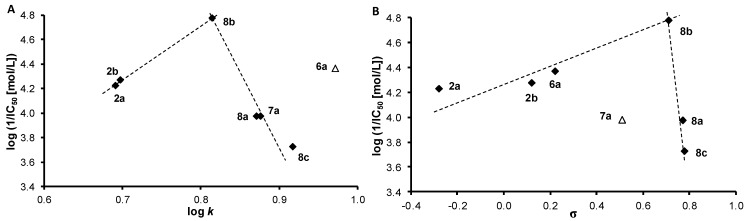
Relationships between PET inhibition log (1/IC_50_) [mol/L] in spinach chloroplasts and lipophilicity expressed as log *k* (**A**) or *N*-substituent electronic Hammett's σ parameters (**B**) of selected studied compounds.

The PET-inhibiting activity was expressed by negative logarithm of IC_50_ value (compound concentration in mol/L causing 50% inhibition of PET). Despite the relatively low inhibitory activity of the studied compounds, correlations between log(1/IC_50_ [mol/L]) and the lipophilicity of compounds expressed as log *k* or electronic properties of individual anilide substituents expressed as Hammett’s σ parameters were performed, see Figure 2. Based on the obtained results it is not possible to decide, whether some of *ortho*-, *meta*- or *para*-positions are preferred from the point of view of PET-inhibiting activity.

The biological activity is affected by lipophilicity (see Table 1 and Figure 2A). In general, when the inactive anilide, *i.e.*, **6a**, was eliminated; the dependence of log (1/IC_50_ [mol/L]) on log *k* is bilinear with the optimum log *k* ca. 0.81 (R = 3-NO_2_, compound **8b**). The corresponding correlation coefficients were r = 0.9993 and r = −0.9673. On the other hand, PET inhibition also increases with electron-withdrawing substituent to σ = 0.71 (R = 3-NO_2_, compound **8b**), where the optimum can be found, and then decreases with a subsequent increase of electron-withdrawing properties of substituents (σ = 0.78, R = 4-NO_2_, compound **8c** and σ = 0.77, R = 2-NO_2_, compound **8a**). Thus, when the activity of compound **7a** is not considered, the dependence of PET-inhibiting activity on the electronic σ properties of substituents (Figure 2B) shows a similar bilinear trend as in case of log *k* (Figure 2A), with correlations coefficients r = 0.9313 (for the σ range from −0.28 to 0.71) and r = −0.9956 for σ > 0.71).

Using artificial electron donors with the known site of action can help in the determination of inhibitory site of action of the studied compound in photosynthetic apparatus. For example, the artificial electron donor 1,5-diphenylcarbazide (DPC) acts in Z^•^/D^•^ intermediate [38] and by supply of electrons it can restore PET in chloroplasts which was inhibited on the donor side of PS II in the section from the oxygen evolving complex to the Z^•^/D^•^ intermediate. However, if PET is inhibited at the acceptor side of PS II, PET will not be restored. Application of the artificial electron donor 1,5-diphenylcarbazide acting in Z^•^/D^•^ intermediate [38] to chloroplasts the activity of which was inhibited by the studied compounds (75% inhibition related to the control) practically completely restored PET (up to 97.8% of the control), indicating that PET on the acceptor side of PS II between the core of PS II (P680) and the secondary plastoquinone molecules/acceptor Q_B_ was not inhibited by the tested compounds. Consequently, it can be assumed that the site of action of the studied compounds is situated on the donor side of PS II in the section between the primary electron donor of PS II (H_2_O) and Z^•^/D^•^ intermediate.

The bilinear course of the dependence of log (1/IC_50_) on σ indicates that for the PET-inhibiting activity not only sufficient lipophilicity (enabling easier penetration of the compounds into the lipids of photosynthetic membranes) but also sufficient electronegativity of the R substituent (enabling interactions with proteins located near oxygen evolving complex present at the luminal side of thylakoid membrane) is necessary. 3-Hydroxy-*N*-(3-nitrophenyl)naphthalene-2-carboxamide (**8b**) was the most active compound from the series, and this result can indicate that PET inhibition can be associated with additional interaction of the nitro moiety with photosynthetic proteins, nevertheless the role of the nitro group in the *meta*-position might originate also from its electron withdrawing properties that influence electron distribution in the aromatic ring. A strong dependence of PET-inhibiting activity on σ was also found for 2-benzylsulphanylbenzimidazoles [39]. The site of action situated on the donor side of PS II was found also for 2-alkylthio-6-R-benzothiazoles (R = 6-formamido-, 6-acetamido-, and 6-benzoylamino-) [40], anilides of 2-alkylpyridine-4-carboxylic acids acting in intermediates Z^•^/D^•^ [41], 5-bromo-*N*-phenylbenzamides [42] and 2-alkylsulphanyl-4-pyridinecarbothioamides acting in the D^•^ intermediate [43].

**Figure 3:**
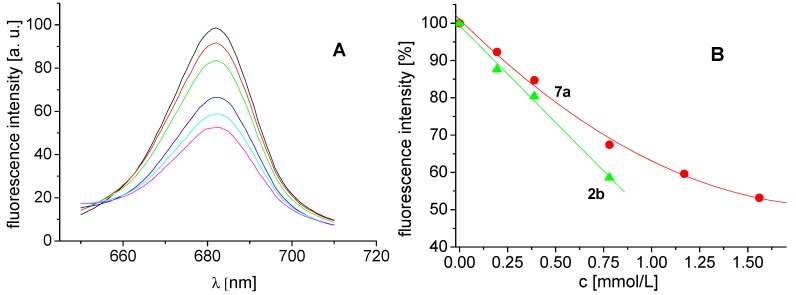
Fluorescence emission spectra of chlorophyll *a* in untreated spinach chloroplasts in presence of compound **7a**: 0, 0.195, 0.390, 0.780, 1.170 and 1.560 mmol/L (curves from top to bottom; λ_ex_ = 436 nm) (**A**) and dependence of fluorescence intensity of chlorophyll *a* on concentration of compounds **2b** (triangles) and **7a** (circles) (**B**).

The effects of the studied compounds on the photosynthetic apparatus of spinach chloroplasts were investigated by studying chlorophyll *a* (Chl*a*) fluorescence. The decreased intensity of the emission band at 686 nm belonging to the pigment-protein complexes in photosystem II [44] (Figure 3A) suggested PS II as the site of action of the studied inhibitors. Lower solubility of compound **2b** did not allow to record fluorescence emission spectra of chloroplasts treated with compound concentration higher than 0.780 mmol/L. The extent of perturbation of chlorophyll *a*-protein complexes in the thylakoid membrane reflected as decreased fluorescence (Figure 3B) correlated with PET inhibiting activity of compounds **2b** and **7a** (IC_50_ = 53.4 and 105.2 mmol/L, respectively). A similar decrease of Chl*a* fluorescence in plant chloroplasts was observed after treatment with HgCl_2_ [45], non-ionic surfactant Triton X 100 [46] as well as substituted benzanilides [47] and salicyanilides [48].

### 2.4. *In Vitro* Antibacterial Susceptibility Testing

Although salicylanilides seem to be promising candidates of antibacterial agents [31,32], all the compounds showed only moderate activity, except for 3-hydroxy-*N*-(2-methoxyphenyl)naphthalene-2-carboxamide (**2a**). As MIC = 55 µmol/L of compound **2a** was the same for all four strains, it can be speculated about specific effectivity against *Staphylococcus* sp. From Table 1 it is obvious that compound **2a** exhibited activity comparable with the standards. 3-Hydroxy-*N*-(2-trifluoromethyl-phenyl)naphthalene-2-carboxamide (**7a**) seems to be another noteworthy compound. Nevertheless due to moderate activity of the rest of the compounds, no thorough SAR could be established.

### 2.5. *In Vitro* Antimycobacterial Evaluation

Although all the compounds were evaluated for their *in vitro* antimycobacterial activity against both mycobacterial strains, most of compounds showed only moderate or no activity, see Table 1. Nevertheless *N*-(2-fluorophenyl)-3-hydroxynaphthalene-2-carboxamide (**4a**) expressed the activity comparable with or higher than the used standard isoniazid against both strains, and 3-hydroxy-*N*-(4-nitrophenyl)naphthalene-2-carboxamide (**8c**) showed relatively high activity against *M. kansasii*.

According to the results, it can be generally concluded that activity against *M. marinum* can be observed especially for *ortho*-substituted anilides. When the least active anilides **3b** and **4c** are eliminated, the dependences of activity on log *k* and on electronic σ properties are bilinear with the optima log *k* ca. 0.73 and σ = 0.06 (R = 2-F, compound **4a**). After the optima the antimycobacterial activity against *M. marinum* decreases with an increase of lipophilicity and/or electron-withdrawing properties of the R substituent.

Within activity against *M. kansasii*, it can be generally concluded that for anilides substituted in *meta*- or *para*-position by methyl and halogens (except the fluoro moiety) the activity slightly increases with increasing lipophilicity and electron-withdrawing properties. For *ortho*-substituted anilides similar bilinear relationships can be found, as discussed above, with the optima log *k* ca. 0.73 and σ = 0.06 (R = 2-F, compound **4a**).

### 2.6. *In Vitro* Cytotoxicity Assay

The most effective compounds **2a**, **2b**, **3b**, **4a**, **7a**, **8b** and **8c** were tested for their *in vitro* cytotoxicity LD_50_ (µmol/L) using human monocytic leukemia THP-1 cells. These cells were used, because they represent human cell line [49]. In the past several works were published, where the toxicity of tested compounds (including antibacterial agents [20,50]) was assessed on THP-1 cells. The highest dose of compounds **2a**,**b** (R = 2-, 3-OCH_3_), **3b** (R = 3-CH_3_) and **4a** (R = 2-F) in the medium (30 μmol/L, it was not possible to solve higher amount) did not lead to significant lethal effect on THP-1 cells, *i.e.*, LD_50_ > 30 µmol/L. Compound **7a** (2-CF_3_) demonstrated low toxicity (LD_50_ = 28.6 ± 0.5 µmol/L) against the human monocytic leukemia THP-1 cell line. Compounds containing 3-NO_2_ (**8b**) and 4-NO_2_ (**8c**) exerted fairly high toxicity LD_50_ = 2.5 ± 0.9 µmol/L for **8b** and LD_50_ < 0.37 µmol/L for **8c** (for example, LD_50_ of oxaliplatin and camptothecin assessed in this line formerly were much lower: 1.7 ± 0.6 µmol/L and 0.20 ± 0.07 µmol/L respectively), which correlates with the positions of these substituents: toxicity increased with the shift of NO_2_ moiety from *ortho* to *para* position (**8b** is less toxic than **8c**). Based on these observations it can be concluded that the discussed amides **2a**, **2b**, **3b** and **4a** can be considered as promising agents for subsequent design of novel antibacterial and antimycobacterial agents, respectively.

## 3. Experimental

### 3.1. General

All reagents were purchased from Aldrich (Sigma-Aldrich, St. Louis, MO, USA). TLC experiments were performed on alumina-backed silica gel 40 F254 plates (Merck, Darmstadt, Germany). The plates were illuminated under UV (254 nm) and evaluated in iodine vapour. The melting points were determined on Kofler hot-plate apparatus HMK (Franz Kustner Nacht KG, Dresden, Germany) and are uncorrected. Infrared (IR) spectra were recorded on a Smart MIRacle™ ATR ZnSe for Nicolet™ Impact 410 FT-IR spectrometer (Thermo Electron Corporation, West Palm Beach, FL, USA). The spectra were obtained by accumulation of 256 scans with 2 cm^−1^ resolution in the region of 4000–600 cm^−1^. All ^1^H- and ^13^C-NMR spectra were recorded on a Bruker Avance III 400 MHz FT-NMR spectrometer (400 MHz for ^1^H and 100 MHz for ^13^C, Bruker Comp., Karlsruhe, Germany). Chemicals shifts are reported in ppm (δ) using internal Si(CH_3_)_4_ as the reference with diffuse, easily exchangeable signals being omitted. Mass spectra were measured using a LTQ Orbitrap Hybrid Mass Spectrometer (Thermo Electron Corporation) with direct injection into an APCI source (400 °C) in the positive mode. The purity of the compounds was checked by the HPLC method using the same conditions as described in Section 3.3. The detection wavelength of 210 nm was chosen. The peaks in the chromatogram of the solvent (blank) were deducted from the peaks in the chromatogram of the sample solution. A purity of individual compounds was determined from area peaks in the chromatogram of the sample solution.

### 3.2. Synthesis

#### 3.2.1. General Procedure for Synthesis of Carboxamide Derivatives **1**–**8c**

3-Hydroxynaphtalene-2-carboxylic acid (1.0 g, 5.3 mmol) was suspended in dry chlorobenzene (30 mL) at ambient temperature and phosphorus trichloride (0.23 mL, 2.7 mmol, 0.5 eq.), and the corresponding substituted aniline (5.3 mmol, 1 eq.) was added dropwise. The reaction mixture was transferred to the microwave reactor, where the synthesis was performed (1st phase: 10 min, 100 °C, 100 W; 2nd phase: 15 min, 120 °C, 500 W; 3rd phase: 20 min, 130 °C, 500 W). Then the mixture was cooled to 60 °C, and then the solvent was removed to dryness under reduced pressure. The residue was washed with hydrochloride acid and water. The crude product was recrystallized from EtOH. Studied compounds **1**–**8c** are presented in Table 1.

*3-Hydroxy-N-phenylnaphthalene-2-carboxamide* (**1**). Yield 76%; Mp. 245–246 °C (Mp. 242–243 °C [34]); HPLC purity 99.41%; IR (Zn/Se ATR, cm^−1^): 3291w, 1620m, 1556m,1494w, 1448w, 1396w, 1344m, 1250w, 1209m, 1173m, 1064w, 950w, 915w, 870m, 842m, 771w, 739s, 712m, 687m; ^1^H-NMR (DMSO-*d*_6_), δ: 11.36 (s, 1H), 10.60 (s, 1H), 8.53 (s, 1H), 7.93 (d *J* = 8.1 Hz, 1H), 7.78 (m, 3H), 7.51 (ddd, *J* = 8.1 Hz, *J* = 6.8 Hz, *J* = 1.3 Hz, 1H), 7.44–7.33 (m, 4H), 7.15 (t, *J* = 7.26 Hz, 1H); ^13^C-NMR (DMSO-*d*_6_), δ: 165.67, 153.79, 138.37, 135.76, 130.42, 128.72, 128.66, 128.08, 126.83, 125.72, 124.03, 123.69, 121.55, 120.55, 110.57; HR-MS: for C_17_H_14_NO_2_[M+H]^+^ calculated 264.1019 *m/z*, found 264.1023 *m/z*.

*3-Hydroxy-N-(2-methoxyphenyl)naphthalene-2-carboxamide* (**2a**). Yield 80%; Mp. 165–166 °C (Mp. 164–166 °C [34]); HPLC purity 99.28%; IR (Zn/Se ATR, cm^−1^): 3191w, 1624m, 1593s, 1549m, 1487w, 1435m, 1393w, 1393w, 1342m, 1284m, 1248w, 1227m, 1173m, 1115m, 1067m, 1031w, 922w, 864m, 837m, 801w, 771m, 735s, 685m; ^1^H-NMR (DMSO-*d*_6_), δ: 11.78 (s, 1H), 11.08 (s, 1H), 8.72 (s, 1H), 8.51 (d, *J* = 7.7 Hz, 1H), 7.98 (d, *J* = 8.1 Hz, 1H), 7.77 (d, *J* = 8.1 Hz, 1H), 7.51 (ddd, *J* = 8.1 Hz, *J* = 6.8 Hz, *J* = 0.9 Hz, 1H), 7.40–7.32 (m, 2H), 7.11–6.92 (m, 3H), 3.92 (s, 3H); ^13^C-NMR (DMSO-*d*_6_), δ: 162.27, 152.61, 148.64, 135.82, 132.50, 128.94, 128.22, 127.91, 127.19, 125.58, 123.94, 123.79, 121.29, 120.62, 120.19, 111.01, 110.71, 56.05; HR-MS: for C_18_H_16_NO_3_ [M+H]^+^ calculated 294.1124 *m/z*, found 294.1130 *m/z*.

*3-Hydroxy-N-(3-methoxyphenyl)naphthalene-2-carboxamide* (**2b**). Yield 75%; Mp. 194–195 °C; HPLC purity 99.05%; IR (Zn/Se ATR, cm^−1^): 3316*w*, 3047w, 1642m, 1622s, 1593s, 1556s, 1456*m*, 1398w, 1365*w*, 1346w, 1266*m*, 1225*m*, 1157*m*, 1134w, 1067m, 1046s, 962w, 913m, 873s, 799*w*, 770m, 752m, 682m; ^1^H-NMR (DMSO-*d*_6_), δ: 11.30 (s, 1H), 10.56 (s, 1H), 8.51 (s, 1H), 7.93 (d, *J* = 8.1 Hz, 1H), 7.77 (d, *J* = 8.1 Hz, 1H), 7.55–7.47 (m, 2H), 7.40–7.24 (m, 4H), 6.76–6.70 (m, 1H), 3.78 (s, 3H); ^13^C-NMR (DMSO-*d*_6_), δ: 165.64, 159.52, 153.67, 139.58, 135.73, 130.42, 129.50, 128.64, 128.06, 126.83, 125.72, 123.69, 121.75, 112.72, 110.54, 109.50, 106.28, 55.03; HR-MS: for C_18_H_16_NO_3_ [M+H]^+^ calculated 294.1124 *m/z*, found 294.1131 *m/z*.

*3-Hydroxy-N-(4-methoxyphenyl)naphthalene-2-carboxamide* (**2c**). Yield 66%; Mp. 235–236 °C; HPLC purity 99.53%; IR (Zn/Se ATR, cm^−1^): 3283w, 3013w, 1636m, 1618s, 1564m, 1510m, 1393w, 1357m, 1303w, 1247m, 1170m, 1146w, 1116w, 1070m, 1030m, 950m, 878m, 856m, 830s, 795w, 775w, 737m; ^1^H-NMR (DMSO-*d*_6_), δ: 11.47 (s, 1H), 10.50 (s, 1H), 8.56 (s, 1H), 7.92 (d, *J* = 7.7 Hz, 1H), 7.76 (d, *J* = 8.6 Hz, 1H), 7.71–7.64 (m, 2H), 7.51 (ddd, *J* = 8.1 Hz, *J* = 6.8 Hz, *J* = 1.3 Hz, 1H), 7.40–7.33 (m, 2H), 7.01–6.93 (m, 2H), 3.77 (s, 3H); ^13^C-NMR (DMSO-*d*_6_), δ: 165.65, 155.91, 154.18, 135.78, 131.25, 130.12, 128.62, 128.06, 126.75, 125.70, 123.64, 122.31, 120.87, 113.87, 110.59, 55.16; HR-MS: for C_18_H_16_NO_3_ [M+H]^+^ calculated 294.1124 *m/z*, found 294.1130 *m/z*.

*3-Hydroxy-N-(2-methylphenyl)naphthalene-2-carboxamide* (**3a**). Yield 74%; Mp. 195–196 °C (Mp. 194–196 °C [34]); HPLC purity 98.84%; IR (Zn/Se ATR, cm^−1^): 3325w, 3115w, 1622s, 1586m, 1548m, 1456m, 1385w, 1355w, 1356w, 1248w, 1173m, 1065m, 954w, 915w, 873m, 844*w*, 803*w*, 773*w*, 742s, 679m; ^1^H-NMR (DMSO-*d*_6_), δ: 11.80 (s, 1H), 10.53 (s, 1H), 8.69 (s, 1H), 7.96 (d, *J* = 7.7 Hz, 2H), 7.78 (d, *J* = 8.1 Hz, 1H), 7.52 (ddd, *J* = 8.1 Hz, *J* = 6.8 Hz, *J* = 1.3 Hz, 1H), 7.41–7.36 (m, 2H), 7.33–7.22 (m, 2H), 7.16–7.08 (m, 1H), 2.34 (s, 3H); ^13^C-NMR (DMSO-*d*_6_), δ: 164,58, 153.57, 136.36, 135.87, 131.45, 130.29, 129.99, 128.82, 128.22, 126.99, 126.24, 125.67, 124.86, 123.76, 123.36, 120.60, 110.72, 17.73; HR-MS: for C_18_H_16_NO_2_ [M+H]^+^ calculated 278.1176 *m/z*, found 278.1182 *m/z*.

*3-Hydroxy-N-(3-methylphenyl)naphthalene-2-carboxamide* (**3b**). Yield 73%; Mp. 207–208 °C; HPLC purity 98.73%; IR (Zn/Se ATR, cm^−1^): 3297w, 3048w, 1607s, 1557 *m*, 1489m, 1451w, 1397w, 1357w, 1344w, 1259m, 1210m, 1173w, 1076w, 951m, 923m, 859s, 836m, 783s, 744s, 689s; ^1^H-NMR (DMSO-*d*_6_), δ: 11.38 (s, 1H), 10.53 (s, 1H), 8.54 (s, 1H), 7.93 (d, *J* = 8.6 Hz, 1H), 7.77 (d, *J* = 8.1 Hz, 1H), 7.60–7.47 (m, 3H), 7.40–7.32 (m, 2H), 7.27 (t, *J* = 7.7 Hz, 1H), 6.97 (d, *J* = 7.3 Hz, 1H), 2.34 (s, 3H); ^13^C-NMR (DMSO-*d*_6_), δ: 165.62, 153.84, 138.26, 137.96, 135.77, 130.42, 128.66, 128.56, 128.08, 126.82, 125.71, 124.74, 123.69, 121.40, 121.06, 117.73, 110.59; 21.11; HR-MS: for C_18_H_16_NO_2_ [M+H]^+^ calculated 278.1176 *m/z*, found 278.1181 *m/z*.

*3-Hydroxy-N-(4-methylphenyl)naphthalene-2-carboxamide* (**3c**). Yield 68%; Mp. 220–221 °C (Mp. 221 °C [35]); HPLC purity 99.56%; IR (Zn/Se ATR, cm^−1^): 3290w, 3008w, 1619s, 1556m, 1516w, 1450w, 1357m, 1252m, 1208m, 1175m, 1121w, 1070m, 951m, 913m, 869s, 832m, 810s, 761w, 741s, 716s; ^1^H-NMR (DMSO-*d*_6_), δ: 11.46 (s, 1H), 10.57 (s, 1H), 8.55 (s, 1H), 7.93 (d, *J* = 8.1 Hz, 1H), 7.77 (d, *J* = 8.1 Hz, 1H), 7.67 (d, *J* = 8.6 Hz, 2H), 7.51 (ddd, *J* = 8.1 Hz, *J* = 6.8 Hz, *J* = 1.3 Hz, 1H), 7.40–7.32 (m, 2H), 7.19 (d, *J* = 8.6 Hz, 2H), 2.29 (s, 3H); ^13^C-NMR (DMSO-*d*_6_), δ: 165.64, 154.01, 135.78, 133.12, 130.32, 129.10, 128.64, 128.06, 126.80, 125.70, 123.66, 121.16, 120.62, 110.60, 20.42; HR-MS: for C_18_H_16_NO_2_ [M+H]^+^ calculated 278.1176 *m/z*, found 278.1182 *m/z*.

*N-(2-Fluorophenyl)-3-hydroxynaphthalene-2-carboxamide* (**4a**). Yield 65%; Mp. 222–223 °C (Mp. 226–228 °C [36]); HPLC purity 97.21%; IR (Zn/Se ATR, cm^−1^): 2981w, 1625s, 1605s, 1556m, 1488w, 1458*m*, 1345m, 1262*m*, 1219*m*, 1206*w*, 1191*w*, 1102*w*, 1065*m*, 1033m, 951*w*, 916e, 867m, 838w, 811*m,* 774w, 740s, 693w; ^1^H-NMR (DMSO-*d*_6_), δ: 11.86 (s, 1H), 10.95 (s, 1H), 8.69 (s, 1H), 8.36 (dt, *J* = 7.7 Hz, *J* = 2.2 Hz, 1H), 7.98 (d, *J* = 8.1 Hz, 1H), 7.78 (d, *J* = 7.7 Hz, 1H), 7.52 (ddd, *J* = 8.1 Hz, *J* = 6.8 Hz, *J* = 1.3 Hz, 1H), 7.41–7.33 (m, 2H), 7.30–7.25 (m, 1H), 7.24–7.16 (m, 2H); ^13^C-NMR (DMSO-*d*_6_), δ: 163.90, 153.08, (d, *J* = 243.8 Hz), 152.88, 135.97, 132.17, 128.93, 128.03, 127.08, 126.27 (d, *J* = 10.7 Hz), 125.66, 124.98 (d, *J* = 6.9 Hz), 124.62 (d, *J* = 3.4 Hz), 123.88, 122.87 (d, *J* = 0.8 Hz), 120.46, 115.18 (d, *J* = 19.1 Hz), 110.81; HR-MS: for C_17_H_13_FNO_2_ [M+H]^+^ calculated 282.0925 *m/z*, found 282.0931 *m/z*.

*N-(3-Fluorophenyl)-3-hydroxynaphthalene-2-carboxamide* (**4b**). Yield 67%; Mp. 248–249 °C; HPLC purity 97.78%; IR (Zn/Se ATR, cm^−1^): 3305w, 3033w, 1622*s*, 1557w, 1516w, 1489w, 1449w, 1401w, 1361w, 1334w, 1253*m*, 1212s, 1178m, 952m, 915m, 872m, 840s 771m, 742s, 715m, 685m; ^1^H-NMR (DMSO-*d*_6_), δ: 11.19(s, 1H), 10.73 (s, 1H), 8.45 (s, 1H), 7.93 (d, *J* = 7.7 Hz, 1H), 7.82 (t, *J* = 2.1 Hz, 1H), 7.79–7.75 (m, 1H), 7.51 (t, *J* = 7.1 Hz, 2H), 7.44–7.32 (m, 3H), 7.02–6.92 (m, 1H); ^13^C-NMR (DMSO-*d*_6_), δ: 165.70, 162.09 (d, *J* = 241.5 Hz), 153.38, 140.24 (d, *J* = 11.1 Hz), 135.73, 130.54, 130.48, 130.30 (d, *J* = 9.5 Hz), 128.63, 128.08, 126.84, 125.73, 123.71, 122.09, 116.06 (d, *J* = 2.7 Hz), 110.32 (d, *J* = 18.7 Hz), 107.10 (d, *J* = 25.9 Hz); HR-MS: for C_17_H_13_FNO_2_ [M+H]^+^ calculated 282.0925 *m/z*, found 282.0932 *m/z*.

*N-(4-Fluorophenyl)-3-hydroxynaphthalene-2-carboxamide* (**4c**). Yield 69%; Mp. 264–265 °C (Mp. 264.5–265.5 °C [36]); HPLC purity 98.03%; IR (Zn/Se ATR, cm^−1^): 3289w, 2994w, 1615s, 1569m, 1505m, 1445w, 1409w, 1357m, 1250w, 1206m, 1171w, 1100m, 1068m, 1011w, 952w, 915m, 871m, 829s, 799m, 766m, 739m, 706m; ^1^H-NMR (DMSO-*d*_6_), δ: 11.31 (s, 1H), 10.64 (s, 1H), 8.49 (s, 1H), 7.93 (d, *J* = 8.12 Hz, 1H), 7.83–7.75 (m, 3H), 7.51 (ddd, *J* = 8.1 Hz, *J* = 6.8 Hz, *J* = 1.3 Hz, 1H), 7.40–7.32 (m, 2H), 7.23 (t, *J* = 9.0 Hz, 2H); ^13^C-NMR (DMSO-*d*_6_), δ: 165.71, 158.50 (d, *J* = 240.7 Hz), 153.79, 135.76, 134.72 (d, *J* = 2.7 Hz), 130.29, 128.62, 128.06, 126.79, 125.72, 123.68, 122.44 (d, *J* = 8.0 Hz), 121.47, 115.28 (d, *J* = 22.1 Hz), 110.55; HR-MS: for C_17_H_13_FNO_2_ [M+H]^+^ calculated 282.0925 *m/z*, found 282.0930 *m/z*.

*N-(2-Chlorophenyl)-3-hydroxynaphthalene-2-carboxamide* (**5a**). Yield 50%; Mp. 226–227 °C (Mp. 225 °C [35]); HPLC purity 98.70%; IR (Zn/Se ATR, cm^−1^): 3164w, 1625m, 1591s, 1546s, 1439m, 1345m, 1293w, 1243w, 1192m, 1171m, 1034m, 840m, 740s, 668m;^1^H-NMR (DMSO-*d*_6_), δ: 11.91 (s, 1H), 11.13 (s, 1H), 8.73 (s, 1H), 8.51 (dd, *J* = 8.1 Hz, *J* = 1.3 Hz, 1H), 7.99 (d, *J* = 7.69 Hz, 1H), 7.78 (d, *J* = 8.12 Hz, 1H), 7.59–7.52 (m, 2H), 7.49–7.45 (m, 1H), 7.41–7.33 (m, 2H), 7.22–7.14 (m, 1H) ^13^C-NMR (DMSO-*d*_6_), δ: 163.49, 152.65, 135.99, 135.29, 132.62, 129.29, 128.99, 128.42, 127.77, 127.13, 125.62, 125.12, 123.88, 123.35, 122.69, 120.55, 110.78; HR-MS: for C_17_H_13_ClNO_2_ [M+H]^+^ calculated 298.0629 *m/z*, found 298.0637 *m/z*.

*N-(3-Chlorophenyl)-3-hydroxynaphthalene-2-carboxamide* (**5b**). Yield 69%; Mp. 257–258 °C (Mp. 258–261 °C [37]); HPLC purity 98.82%; IR (Zn/Se ATR, cm^−1^): 3299w, 3054w, 1623s, 1545m, 1476w, 1426m 1364m, 1278w, 1248m, 1214m, 1174w, 1065w, 913w, 866m, 776m, 744m, 708m, 680m; ^1^H-NMR (DMSO-*d*_6_), δ: 11.16 (s, 1H); 10.68 (s, 1H), 8.46 (s, 1H), 7.99 (t, *J* = 1.9 Hz, 1H), 7.93 (d, *J* = 8.1 Hz, 1H), 7.76 (d, *J* = 7.7 Hz, 1H), 7.67 (d, *J* = 8.1 Hz, 1H), 7.51 (ddd, *J* = 8.1 Hz, *J* = 6.8 Hz, *J* = 1.3 Hz, 1H), 7.47–7.34 (m, 3H), 7.2 (d, *J* = 8.1 Hz, 1H); ^13^C-NMR (DMSO-*d*_6_), δ: 165.77, 153.42, 139.95, 135.73, 133.06, 130.45, 130.35, 128.61, 128.09, 126.81, 125.73, 123.71, 123.60, 122.03, 119.80, 118.73, 110.52; HR-MS: for C_17_H_13_ClNO_2_ [M+H]^+^ calculated 298.0629 *m/z*, found 298.0637 *m/z*.

*N-(4-Chlorophenyl)-3-hydroxynaphthalene-2-carboxamide* (**5c**). Yield 71%; Mp. 263–264 °C (Mp. 260 °C [34]); HPLC purity 99.35%; IR (Zn/Se ATR, cm^−1^): 3283w, 3052w, 1612s, 1549m, 1488m, 1400m, 1359w, 1334w, 1252w, 1209m, 1170m, 1116w, 1068w, 1012m, 749s, 711m, 680m;^1^H-NMR (DMSO-*d*_6_), δ: 11.24 (s, 1H), 10.68 (s, 1H), 8.47 (s, 1H), 7.93 (d, *J* = 7.7 Hz, 1H), 7.8 (t, *J* = 9.6 Hz, 2H), 7.54–7.46 (m, 2H), 7.42–7.32 (m, 4H); ^13^C-NMR (DMSO-*d*_6_), δ: 165.69, 153.56, 137.42, 135.74, 130.45, 130.40, 128.61, 128.08, 127.61, 126.81, 125.73, 123.70, 121.97, 121.83, 110.53; HR-MS: for C_17_H_13_ClNO_2_ [M+H]^+^ calculated 298.0629 *m/z*, found 298.0636 *m/z*.

*N-(2-Bromophenyl)-3-hydroxynaphthalene-2-carboxamide* (**6a**). Yield 61%; Mp. 215–216 °C; HPLC purity 97.75%; IR (Zn/Se ATR, cm^−1^): 3158w, 1623s, 1582s, 1537s, 1446w, 1434m, 1388w, 1343*m*, 1312w, 1291*w*, 1193*w*, 1071w, 1047*w*, 1024*m*, 916m, 873m, 845m, 750s, 698*m*; ^1^H-NMR (DMSO-*d*_6_), δ: 11.88 (s, 1H), 11.00 (s, 1H), 8.72 (s, 1H), 8.42 (dd, *J* = 8.1 Hz, *J* = 1.3 Hz, 1H), 7.98 (d, *J* = 8.1 Hz 1H), 7.80–7.70 (m, 2H), 7.57–7.50 (m, 2H), 7.45–7.34 (m, 2H), 7.13 (dt *J* = 7.7 Hz, *J* = 1.7 Hz, 1H); ^13^C-NMR (DMSO-*d*_6_), δ: 163.67, 152.77, 136.53, 136.02, 132.59, 132.54, 128.99, 128.45, 128.28, 127.10, 125.82, 125.64, 123.88, 123.50, 120.47, 114.35, 110.76; HR-MS: for C_17_H_13_BrNO_2_ [M+H]^+^ calculated 342.0124 *m/z*, found 342.0133 *m/z*.

*N-(3-Bromophenyl)-3-hydroxynaphthalene-2-carboxamide* (**6b**). Yield 73%; Mp. 251–252 °C; HPLC purity 98.67%; IR (Zn/Se ATR, cm^−1^): 3295w, 3075w, 1622*m*, 1583*m*, 1553w, 1479m, 1403w, 1239*w*, 1209*m*, 1095*w*, 1073*w*, 992w, 953w, 923w, 867m 852s, 837m, 780s, 745s, 667m; ^1^H-NMR (DMSO-*d*_6_), δ: 11.16 (s, 1H), 10.00 (s, 1H), 8.46 (s, 1H), 8.13 (s, 1H), 7.92 (d, *J* = 7.69 Hz, 1H), 7.78-7.68 (m, 2H), 7.51 (ddd, *J* = 8.1 Hz, *J* = 6.8 Hz, *J* = 1.3 Hz, 1H), 7.40–7.33 (m, 4H); ^13^C-NMR (DMSO-*d*_6_), δ: 165.79, 153.44, 140.08, 135.74, 130.66, 130.43, 128.62, 128.09, 126.80, 126.51, 125.74, 123.72, 122.66, 122.00, 121.48, 119.14, 110.52; HR-MS: for C_17_H_13_BrNO_2_ [M+H]^+^ calculated 342.0124*m/z*, found 342.0130 *m/z*.

*N-(4-Bromophenyl)-3-hydroxynaphthalene-2-carboxamide* (**6c**). Yield 69%; Mp. 253–254 °C (Mp. 248–249 °C [35]); HPLC purity 99.02%; IR (Zn/Se ATR, cm^−1^): 3288w, 3052w, 1605*s*, 1544*m*, 1486*s*, 1447w, 1394m, 1360w, 1251*w*, 1209*m*, 1169*w*, 1075*m*, 1010m, 955*w*, 915w, 871m, 828*s*, 810s, 785m, 749s, 712m; ^1^H-NMR (DMSO-*d*_6_), δ: 11.22 (s, 1H), 10.67 (s, 1H), 8.46 (s, 1H) 7.92 (d, *J* = 7.7 Hz, 1H), 7.76 (d, *J* = 8.6 Hz, 3H), 7.59–7.46 (m, 3H), 7.39–7.32 (m, 2H); ^13^C-NMR (DMSO-*d*_6_), δ: 165.66, 153.51, 137.83, 135.72, 131.52, 130.39, 128.60, 128.06, 126.80, 125.70, 123.69, 122.30, 121.87, 115.63, 110.52; HR-MS: for C_17_H_13_BrNO_2_ [M+H]^+^ calculated 342.0124*m/z*, found 342.0132 *m/z*.

*3-Hydroxy-N-(2-trifluoromethylphenyl)naphthalene-2-carboxamide* (**7a**). Yield 49%; Mp. 209–210 °C; HPLC purity 99.66%; IR (Zn/Se ATR, cm^−1^): 3274w, 1660w, 1626w, 1597w, 1515m, 1466s, 1352w, 1278s, 1217w, 1198*w*, 1146m, 1071w, 956*m*, 874*m*, 833*m*, 788*m*, 748*w*, 722m; ^1^H-NMR (DMSO-*d*_6_), δ: 11.67 (s, 1H), 10.43 (s, 1H), 8.72 (s, 1H), 7.99 (d, *J* = 8.1 Hz, 1H), 7.86 (d, *J* = 7.8 Hz, 1H), 7.83–7.77 (m, 2H), 7.70–7.66 (m, 1H), 7.62–7.57 (m, 1H), 7.53 (ddd, *J* = 7.0 Hz, *J* = 5.5 Hz, *J* = 1.1 Hz, 1H), 7.40 (s, 1H), 7.36 (ddd, *J* = 8.4 Hz, *J* = 7.0 Hz, *J* = 1.1 Hz); ^13^C-NMR (DMSO-*d*_6_), δ: 167.12, 153.35, 135.78, 135.47 (q, *J* = 3.9 Hz), 133.15, 131.16, 130.42, 128.63, 128.12, 126.86, 126.45 (q, *J* = 4.6 Hz), 126.16 (q, *J* = 29.6 Hz), 125.75, 123.75, 123.70, (q, J = 276.2 Hz), 122.11, 120.19, 110.55; HR-MS: for C_18_H_13_NO_2_F_3_ [M+H]^+^ calculated 332.0893 *m/z*, found 332.0898 *m/z*.

*3-Hydroxy-N-(3-trifluoromethylphenyl)naphthalene-2-carboxamide* (**7b**). Yield 64%; Mp. 239–240 °C; HPLC purity 98.81%; IR (Zn/Se ATR, cm^−1^): 3296w, 3108w, 1626s, 1575m, 1494m, 1428w, 1399w, 1361w, 1327s, 1207m, 1178s, 1148m, 1118s, 1100m, 1076m, 925w, 893m, 867s, 840m, 803s, 771m, 750s, 696s, 680m; ^1^H-NMR (DMSO-*d*_6_), δ: 11.16 (s, 1H), 10.83 (s, 1H), 8.45 (s, 1H), 8.29 (s, 1H), 7.96 (t, *J* = 8.1 Hz, 2H), 7.97 (d, *J* = 8.1 Hz, 1H), 7.62 (t, *J* = 7.9 Hz, 1H), 7.55–7.47 (m, 2H), 7.40–7.32 (m, 2H); ^13^C-NMR (DMSO-*d*_6_), δ: 166.06, 153.46, 139.32, 135.79, 130.45, 129.93, 129.51 (q, *J* = 31.3 Hz), 128.63, 128.13, 126.84, 125.76, 124.11 (q, *J* = 277.7 Hz), 123.94, 123.76, 122.09, 120.21 (q, *J* = 3.8 Hz), 116.47 (q, *J* = 3.8 Hz), 110.54; HR-MS: for C_18_H_13_NO_2_F_3_ [M+H]^+^ calculated 332.0893 *m/z*, found 332.0900 *m/z*.

*3-Hydroxy-N-(4-trifluoromethylphenyl)naphthalene-2-carboxamide* (**7c**). Yield 58%; Mp. 281–282 °C; HPLC purity 99.46%; IR (Zn/Se ATR, cm^−1^): 3292w, 3021w, 1623s, 1548m, 1450m, 1410m, 1324m, 1255w, 1212m, 1175m, 1112s, 1065s, 1016m, 960w, 916w, 873m, 841s, 822m, 791w, 752s, 707m; ^1^H-NMR (DMSO-*d*_6_), δ: 11.17 (s, 1H), 10.85 (s, 1H), 8.45 (s, 1H), 8.03–7.91 (m, 3H), 7.79–7.72 (m, 3H), 7.51 (ddd, *J* = 8.1 Hz, *J* = 6.8 Hz, *J* = 1.3 Hz, 1H), 7.40–7.32 (m, 2H); ^13^C-NMR (DMSO-*d*_6_), δ: 165.87, 153.28, 142.15 (q, *J* = 1.5 Hz), 135.76, 130.61, 128.64, 128.12, 126.86, 125.98 (q, *J* = 3.8 Hz), 125.74, 124.30 (q, *J* = 271.2Hz), 123.88 (q, *J* = 32.0 Hz), 123.74, 122.28, 120.18, 110.53; HR-MS: for C_18_H_13_NO_2_F_3_ [M+H]^+^ calculated 332.0893 *m/z*, found 332.0899 *m/z*.

*3-Hydroxy-N-(2-nitrophenyl)naphthalene-2-carboxamide* (**8a**). Yield 29%; Mp. 174–175 °C; HPLC purity 97.17%; IR (Zn/Se ATR, cm^−1^): 3240w, 1627m,1581m, 1557w, 1494m, 1450w, 1434w, 1393w, 1341m, 1270m, 1203w, 1147m, 870w, 840m, 771w, 736s, 691w; ^1^H-NMR (DMSO-*d*_6_), δ: 12.05 (s, 1H), 11.79 (s, 1H), 8.73 (s, 1H), 8.60 (dd, *J* = 7.2 Hz, *J* = 1.2 Hz, 1H), 8.16 (dd, *J* = 8.2 Hz, *J* = 1.5 Hz, 1H), 7.97 (t, *J* = 8.0 Hz, 2H), 7.82–7.78 (m, 1H), 7.51 (ddd, *J* = 8.1 Hz, *J* = 6.8 Hz, *J* = 1.3 Hz, 1H), 7.39–7.34 (m, 3H); ^13^C-NMR (DMSO-*d*_6_), δ: 164.21, 155.98, 152.85, 137.18, 136.17, 132.97, 132.43, 129.16, 128.94, 127.04, 126.62, 125.89, 125.63, 123.75, 120.74, 115.27, 110,75; HR-MS: for C_17_H_13_N_2_O_4_ [M+H]^+^ calculated 309.0870 *m/z*, found 309.0875 *m/z*.

*3-Hydroxy-N-(3-nitrophenyl)naphthalene-2-carboxamide* (**8b**). Yield 58%; Mp. 250–251 °C; (Mp. 242–244 °C [34]); HPLC purity 97.62%; IR (Zn/Se ATR, cm^−1^): 3394w, 1658m, 1586m, 1525m, 1463m, 1345s, 1297s, 1230m, 1209m, 1145m, 1033w, 911w, 876m, 808m, 747m, 738s, 668m; ^1^H-NMR (DMSO-*d*_6_), δ: 11.12 (s, 1H), 10.93 (s, 1H), 8.83 (t, *J* = 2.14 Hz, 1H), 8.45 (s, 1H), 8.11 (dd, *J* = 8.12 Hz, *J* = 1.28 Hz, 1H), 8.01–7.91 (m, 2H), 7.77 (d, *J* = 8.1 Hz, 1H), 7.66 (t, *J* = 8.1 Hz, 1H), 7.51 (ddd, *J* = 8.1 Hz, *J* = 6.8 Hz, *J* = 1.3 Hz, 1H), 7.40–7.32 (m, 2H); ^13^C-NMR (DMSO-*d*_6_), δ: 166.08, 153.30, 147.95, 139.72, 135.76, 130.48, 130.08, 128.61, 128.12, 126.80, 126.24, 125.76, 123.76, 122.22, 118.32, 114.35, 110.50; HR-MS: for C_17_H_13_N_2_O_4_ [M+H]^+^ calculated 309.0870 *m/z*, found 309.0876 *m/z*.

*3-Hydroxy-N-(4-nitrophenyl)naphthalene-2-carboxamide* (**8c**). Yield 72%; Mp. 265–266 °C; HPLC purity 99.03%; IR (Zn/Se ATR, cm^−1^): 3285w, 2981w, 1618s, 1599m, 1568w, 1513s, 1449w, 1407w, 1337s, 1260w, 1210m, 1171m, 1147w, 1112m, 1033m, 916w, 851s, 772w, 747s, 704w; ^1^H-NMR (DMSO-*d*_6_), δ: 11.10 (s, 1H), 11.03 (s, 1H), 8.41 (s, 1H), 8.28 (d, *J* = 9.4 Hz, 2H), 8.03 (d, *J* = 9.0 Hz, 2H), 7.93 (d, *J* = 8.1Hz, 1H), 7.77 (d, *J* = 8.6 Hz, 1H), 7.51 (ddd, *J* = 8.1 Hz, *J* = 6.8 Hz, *J* = 1.3 Hz, 1H), 7.39–7.32 (m, 2H); ^13^C-NMR (DMSO-*d*_6_), δ: 165.89, 152.98, 144.80, 142.58, 135.76, 130.73, 128.63, 128.15, 126.85, 125.75, 124.82, 123.76, 122.68, 119.79, 110.49; HR-MS: for C_17_H_13_N_2_O_4_ [M+H]^+^ calculated 309.0870 *m/z*, found 309.0875 *m/z*.

### 3.3. Lipophilicity Determination by HPLC (Capacity Factor k/Calculated log k)

A HPLC system Agilent 1200 equipped with DAD detector (Agilent, Santa Clara, CA, USA) was used. A chromatographic column Symmetry^®^ C_18_ 5 μm, 4.6 × 250 mm, Part No. WAT054275, (Waters Corp., Milford, MA, USA) was used. The HPLC separation process was monitored and evaluated by EZChrom Elite software ver. 3.3.2 (Agilent). Isocratic elution by a mixture of MeOH p.a. (60%) and H_2_O-HPLC Mili-Q grade (40%) as a mobile phase was used. The total flow of the column was 1.0 mL/min, injection 20 μL, column temperature 40 °C and sample temperature 10 °C. The detection wavelength 210 nm was chosen. The KI methanolic solution was used for the dead time (t_D_) determination. Retention times (t_R_) were measured in minutes. The capacity factors *k* were calculated according to formula *k* = (t_R_ − t_D_)/t_D_, where t_R_ is the retention time of the solute, whereas t_D_ denotes the dead time obtained using an unretained analyte. Log *k*, calculated from the capacity factor *k*, is used as the lipophilicity index converted to log *P* scale. The log *k* values of the individual compounds are shown in Table 1.

### 3.4. Study of Inhibition of Photosynthetic Electron Transport (PET) in Spinach Chloroplasts

Chloroplasts were prepared from spinach (*Spinacia oleracea* L.) according to Masarovicova and Kralova [51]. The inhibition of photosynthetic electron transport (PET) in spinach chloroplasts was determined spectrophotometrically (Genesys 6, Thermo Scientific), using an artificial electron acceptor 2,6-dichlorophenol-indophenol (DCIPP) according to Kralova *et al*. [52], and the rate of photosynthetic electron transport was monitored as a photoreduction of DCPIP. The measurements were carried out in phosphate buffer (0.02 mol/L, pH 7.2) containing sucrose (0.4 mol/L), MgCl_2_ (0.005 mol/L) and NaCl (0.015 mol/L). The chlorophyll content was 30 mg/L in these experiments and the samples were irradiated (~100 W/m^2^ with 10 cm distance) with a halogen lamp (250 W) using a 4 cm water filter to prevent warming of the samples (suspension temperature 22 °C). The studied compounds were dissolved in DMSO due to their limited water solubility. The applied DMSO concentration (up to 4%) did not affect the photochemical activity in spinach chloroplasts. The inhibitory efficiency of the studied compounds was expressed by IC_50_ values, *i.e.*, by molar concentration of the compounds causing 50% decrease in the oxygen evolution rate relative to the untreated control. The comparable IC_50_ value for a selective herbicide 3-(3,4-dichlorophenyl)-1,1-dimethylurea, DCMU (Diurone^®^) was about 1.9 μmol/L. The results are summarized in Table 1.

### 3.5. Study of Chlorophyll a Fluorescence in Spinach Chloroplasts

The fluorescence emission spectra of chlorophyll *a* (Chl*a*) in spinach chloroplasts were recorded on fluorescence spectrophotometer F-2000 (Hitachi, Tokyo, Japan) using excitation wavelength λ_ex_ = 436 nm for monitoring fluorescence of Chl*a*, excitation slit 20 nm and emission slit 10 nm. The samples were kept in the dark for 2 min before measuring. The phosphate buffer used for dilution of the chloroplast suspension was the same as described above. Due to low aqueous solubility the compounds were added to chloroplast suspension in DMSO solution. The DMSO concentration in all samples was the same as in the control (10%). The chlorophyll concentration in chloroplast suspension was 10 mg/L.

### 3.6. *In Vitro* Antibacterial Susceptibility Testing

The synthesized compounds were evaluated for *in vitro* antibacterial activity against representatives of multidrug-resistant bacteria, clinical isolates of methicillin-resistant *Staphylococcus aureus* (MRSA) 63718, SA 630 and SA 3202 that were obtained from the National Institute of Public Health, Prague, Czech Republic. *Staphylococcus aureus* ATCC 29213 was used as a reference and quality control strain. Ampicillin (Sigma-Aldrich) was used as the standard. Prior to testing, each strain was passaged onto nutrient agar (Oxoid, Hampshire, UK) with 5% of bovine blood, and bacterial inocula were prepared by suspending a small portion of bacterial colony in sterile phosphate buffered saline (pH 7.2–7.3). The cell density was adjusted to 0.5 McFarland units using a densitometer (Densi-La-Meter, LIAP, Riga, Latvia). The final inoculum was made by 1:20 dilution of the suspension with the Mueller-Hinton broth (MH broth). The compounds were dissolved in DMSO (Sigma), and the final concentration of DMSO in the MH broth (Oxoid) did not exceed 2.5% of the total solution composition. The broth dilution micro-method modified according to NCCLS guidelines [53,54] in MH broth was used to determine the minimum inhibitory concentration (MIC). Drug-free controls, sterility controls and controls consisted of MH broth and DMSO alone were included. The determination of results was performed visually after 24 h of static incubation in the darkness at 37 °C in an aerobic atmosphere. The MICs were defined as the lowest concentration of the compound at which no visible bacterial growth was observed. The results are summarized in Table 1.

### 3.7. *In Vitro* Antimycobacterial Evaluation

The evaluation of *in vitro* antimycobacterial activity of the compounds was performed against *Mycobacterium marinum* CAMP 5644 and *M. kansasii* DSM 44162. The broth dilution micro-method in Middlebrook 7H9 medium (Difco, Lawrence, KS, USA) supplemented with ADC Enrichment (BBL, USA) was used to determine the minimum inhibitory concentration (MIC) as previously described [55]. The tested compounds were dissolved as described above in chapter 3.6. Isoniazid (Sigma-Aldrich) was used as reference antibacterial drug. Bacterial inocula were prepared by transferring colonies from culture to sterile water. The cell density was adjusted to 0.5 McFarland units using a densitometer (Densi-La-Meter, LIAP). The final inoculum was made by 1:1,000 dilution of the suspension with sterile water. Drug-free controls, sterility controls and controls consisted of medium and DMSO alone were included. The determination of results was performed visually after 7 days of static incubation in the darkness at 37 °C in an aerobic atmosphere for *M. kansasii* and after 21 days of static incubation in the darkness at 28 °C in an aerobic atmosphere for *M. marinum*. The MICs were defined as the lowest concentration of the compound at which no visible bacterial growth was observed. The results are summarized in Table 1.

### 3.8. *In Vitro* Cytotoxicity Assay

Human monocytic leukemia THP-1 cells were used for *in vitro* toxicity assay. Cells were obtained from the European Collection of Cell Cultures (ECACC, Salisbury, UK) and routinely cultured in RPMI medium supplemented with 10% fetal bovine serum, 2% l-glutamine, 1% penicillin and streptomycin at 37 °C with 5% CO_2_. Cytotoxicity was determined using a WST-1 assay kit (Roche Diagnostics, Mannheim, Germany) according to the manufacturer’s instructions. The tested compounds were dissolved in DMSO and added in five increasing concentrations (0.37, 1.1, 3.3, 10, and 30 μmol/L) to the cell suspension in the culture medium. Subsequently, the cells were incubated for 24 h at 37 °C with 5% CO_2_. WST-1 assays conducted in triplicates were performed as previously described [49]. The median lethal dose values, LD_50_, were deduced through the production of a dose-response curve. All data were evaluated using GraphPad Prism 5.00 software (GraphPad Software, San Diego, CA, USA). The results are summarized in Table 1.

## 4. Conclusions

A series of twenty-two ring-substituted 3-hydroxynaphthalene-2-carboxanilides were prepared and characterized. The prepared compounds were tested for their ability to inhibit photosynthetic electron transport (PET) in spinach chloroplasts (*Spinacia oleracea* L.) and for their antibacterial and antimycobacterial activity against four *Staphylococcus* strains, *Mycobacterium marinum* and *M. kansasii*. 3-Hydroxy-*N*-(3-nitrophenyl)naphthalene-2-carboxamide (**8b**) showed relatively high PET inhibition. 3-Hydroxy-*N*-(2-methoxyphenyl)naphthalene-2-carboxamide (**2a**) showed the high biological activity against *S. aureus* as well as methicillin-resistant strains. *N*-(2-Fluorophenyl)-3-hydroxynaphthalene-2-carboxamide (**4a**) and *N*-(3-fluorophenyl)-3-hydroxynaphthalene-2-carboxamide (**4b**) expressed high activity against *M. marinum*. 3-Hydroxy-*N*-(4-nitrophenyl)naphthalene-2-carboxamide (**8c**) showed high activity against *M. kansasii*. All the above-mentioned compounds exhibited activity comparable with or higher than the standards ampicillin or isoniazid. Lipophilicity and electronic properties of anilide substituents influenced the biological activities of compounds in all biological assays. It can be stated that the dependences of activities on lipophilicity and on electronic properties show bilinear trends. The most effective compounds **2a**, **4a**, **7a**, **8b** and **8c** were tested for their *in vitro* cytotoxicity against THP-1 cells. It can be concluded that the discussed anilides **2a** and **4a** can be considered as promising agents for subsequent design of novel antibacterial and antimycobacterial agents.
